# Vaccine effectiveness estimates from an early-season influenza A(H3N2) epidemic, including unique genetic diversity with reassortment, Canada, 2022/23

**DOI:** 10.2807/1560-7917.ES.2023.28.5.2300043

**Published:** 2023-02-02

**Authors:** Danuta M Skowronski, Erica SY Chuang, Suzana Sabaiduc, Samantha E Kaweski, Shinhye Kim, James A Dickinson, Romy Olsha, Jonathan B Gubbay, Nathan Zelyas, Hugues Charest, Nathalie Bastien, Agatha N Jassem, Gaston De Serres

**Affiliations:** 1 British Columbia Centre for Disease Control, Vancouver, Canada; 2 University of British Columbia, Vancouver, Canada; 3 University of Calgary, Calgary, Canada; 4 Public Health Ontario, Toronto, Canada; 5 University of Toronto, Toronto, Canada; 6 Public Health Laboratory, Alberta Precision Laboratories, Edmonton, Canada; 7 Institut National de Santé Publique du Québec, Québec, Canada; 8 National Microbiology Laboratory, Public Health Agency of Canada, Winnipeg, Canada; 9 Laval University, Quebec, Canada; 10 Centre Hospitalier Universitaire de Québec, Québec, Canada

**Keywords:** Influenza, A(H3N2), vaccine effectiveness, clade, test-negative design, observational study, reassortment, imprinting

## Abstract

The Canadian Sentinel Practitioner Surveillance Network estimated vaccine effectiveness (VE) during the unusually early 2022/23 influenza A(H3N2) epidemic. Like vaccine, circulating viruses were clade 3C.2a1b.2a.2, but with genetic diversity affecting haemagglutinin positions 135 and 156, and reassortment such that H156 viruses acquired neuraminidase from clade 3C.2a1b.1a. Vaccine provided substantial protection with A(H3N2) VE of 54% (95% CI: 38 to 66) overall. VE was similar against H156 and vaccine-like S156 viruses, but with potential variation based on diversity at position 135.

During the 2021/22 season, Canada experienced a delayed influenza A(H3N2) epidemic caused by clade 3C.2a1b.2a.2 (‘2a.2’) viruses that peaked in May 2022 (week 19) [1,2]. Thereafter, Canada [3,4], like other countries of Europe and the United States (US) [5,6], experienced an early 2022/23 A(H3N2) epidemic, also caused by 2a.2 viruses, that began in late October 2022 (week 43), peaked in November (weeks 47–48) and subsided by January 2023 (week 1) [3,4]. The Canadian Sentinel Practitioner Surveillance Network (SPSN) aimed to assess 2022/23 vaccine effectiveness (VE) against influenza A(H3N2), including genetic characterisation of contributing viruses for context- and variant-specific comparison.

## Vaccine effectiveness evaluation

Influenza VE was estimated by test-negative design. Eligible patients aged ≥ 1 year presented within 7 days of influenza-like-illness onset to community-based sentinel practitioners in Alberta, British Columbia (BC), Ontario and Quebec [1,7,8]. Analyses included nasal or nasopharyngeal specimens collected from eligible patients between 1 November 2022 (week 44) and 6 January 2023 (week 1), tested for influenza by real-time RT-PCR and/or multiplex assays. Influenza vaccination status was based on self-report. In SPSN provinces, > 99% of publicly funded vaccines were egg-based inactivated. Elderly adults ≥ 65 years were administered MF59-adjuvanted or high-dose vaccines [9], the latter including facility residents in BC and Quebec but all elderly adults in Ontario and Alberta.

The 2022/23 influenza A(H3N2) vaccine was updated from the 2021/22 clade 3C.2a1b.2a.1 to a 2a.2 strain [10]. The representative 2a.2 anchor strain was A/Bangladesh/4005/2020 but vaccines used A/Darwin/6/2021 (cell-passaged) and A/Darwin/9/2021 (egg-passaged) 2a.2 reference viruses [10-12]. Like virtually all A(H3N2) viruses since 2002, A/Bangladesh has H156 in the haemagglutinin (HA) whereas A/Darwin reflects recent 2a.2 viruses in 2021 and 2022 with an H156S substitution (i.e. A/Darwin vaccines are S156) [10-14].

## Virological evaluation

Sanger sequencing of the HA gene was undertaken on a convenience sample of original patient specimens. Whole genome sequencing (WGS), adapted from published protocols [15-18], was applied to a further subset to characterise the neuraminidase (NA). Viruses were classified per recent nomenclature from the European Centre for Disease Prevention and Control (ECDC) as genetic subgroups i–iv; for cross reference, current Nextstrain terminology is as follows for corresponding ECDC subgroups i (‘2b’), ii (‘2a.3a.1’), iii (‘2a.1’) and iv (‘2a.1b’) [12,14]. We indicate HA amino acid substitutions in relation to A/Bangladesh, including antigenic sites in parentheses, and annotate involvement of the receptor-binding site (RBS) and potential gain or loss of N-linked glycosylation (+/− CHO) [19,20]. In Supplementary Table S1 we provide detailed sequencing findings, including additional HA substitutions and NA characterisation. For the 387 viruses with sufficient sequence quality for upload, data are available in the Global Initiative on Sharing All Influenza Data (GISAID) database with GISAID identification numbers: EPI_ISL_16755416 to EPI_ISL_16755802 [21].

## Virological findings

**Figure 1:**
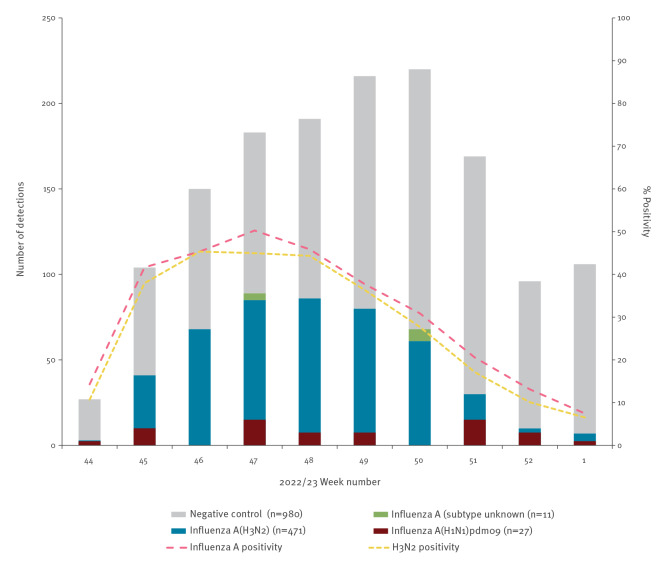
Influenza detections among eligible patients presenting with influenza-like illness, by week of specimen collection, SPSN, Canada, 1 November 2022–6 January 2023 (weeks 44–1) (n = 1,490)

Among 1,490 eligible specimens, 510 (34%) tested influenza-positive, including 509 influenza A and one influenza B (Figure 1). Of the 498 (98%) subtyped influenza A viruses, 471 (95%) were A(H3N2). The HA of 391 (83%) A(H3N2) viruses was genetically characterised, including 89 of 97 (92%), 62 of 77 (81%), 163 of 213 (77%) and 77 of 84 (92%) from BC, Alberta, Ontario and Quebec, respectively.

SPSN: Sentinel Practitioner Surveillance Network.

Among eligible patients presenting with influenza-like illness, influenza test-positive cases and test-negative controls are displayed by week of specimen collection in 2022/23. Tallies correspond with participant profiles shown below but with influenza A(H1N1)pdm09 and unsubtyped influenza A detections additionally displayed. Not displayed is a single influenza B detection in week 49. The figure excludes cases and controls vaccinated within 2 weeks of illness onset and those with missing information for covariates used in primary vaccine effectiveness analyses. As per usual SPSN approach, missing specimen collection dates were imputed as the date the specimen was received and processed at the laboratory minus 2 days.

**Figure 2:**
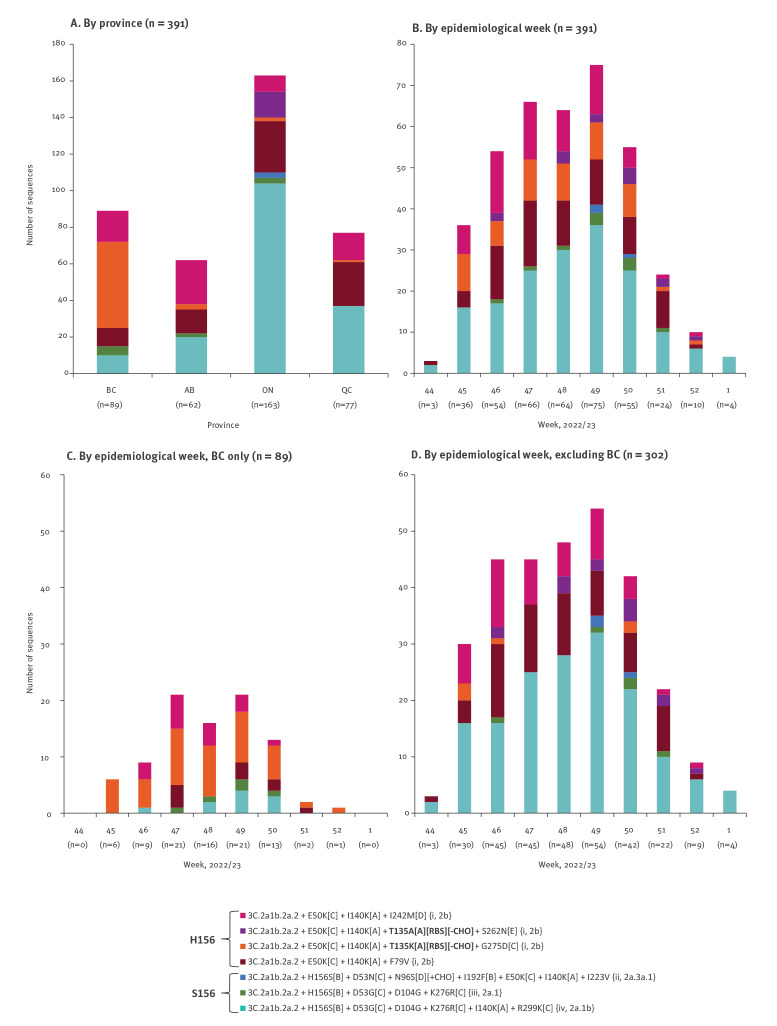
Distribution of clade 3C.2a1b.2a2 variants among genetically characterised influenza A(H3N2) viruses contributing to influenza vaccine effectiveness analyses, SPSN, Canada, 1 November–28 December 2022 (weeks 44–1) (n = 391)

BC: British Columbia; CHO: carbohydrate; ECDC: European Centre for Disease Prevention and Control; RBS: receptor-binding site; SPSN: Sentinel Practitioner Surveillance Network.

Supplementary Table S1 provides detailed gene sequencing findings. All genetically characterised viruses were clade 2a.2, with 207 of 391 (53%) belonging to ECDC subgroup i, defined by H156 with E50K(C) (Figure 2). In BC, most characterised viruses (83%; 74/89) belonged to the ECDC subgroup i, with most of these (64%; 47/74) bearing additional T135K(A)(RBS)(−CHO) substitution. In other SPSN provinces, less than half (44%; 133/302) of characterised viruses belonged to subgroup i, of which few (9%; 6/133) bore the additional T135K substitution. Of note, position 135 is an accessory site previously involved in the major antigenic cluster transition to A/Sydney/5/97-like viruses in 1997 [13]. Prior to that, K135 viruses had briefly predominated between 1993 and 1996, but with K135T(A)(RBS)(+CHO) substitution accompanying A/Sydney emergence, T135 viruses have predominated since [21,22]. During the 2022/23 epidemic, H156 viruses with T135K substitution comprised more than half (53%; 47/89) of characterised viruses in BC, but < 5% in other SPSN provinces (2%; 6/302). In Ontario, 14 of 163 (9%) characterised viruses were instead H156 with T135A(A)(RBS)(−CHO), a substitution not found in other SPSN provinces.

−CHO and +CHO refer to mutations resulting in the loss or gain of a potential glycosylation site, respectively. Genetically characterised influenza A(H3N2) variants contributing to influenza vaccine effectiveness analyses with associated substitutions and ECDC subgroup designations, followed by Nextstrain nomenclature, noted in curly brackets. Colour coding corresponds with subgroups displayed in Supplementary Table S1, the latter including further substitutions and neuraminidase characterisation.

The remaining 184 of 391 (47%) SPSN viruses not belonging to subgroup i were H156S(B) (i.e. S156, like the A/Darwin vaccines) with additional substitutions placing them within ECDC subgroups ii (< 1%; 3/391), iii (3%; 10/391) or iv (44%; 171/391), the latter S156 viruses including two also bearing T135K substitution (Figure 2). 

Among SPSN viruses subjected to WGS (33%; 154/471), we additionally identified a reassortment combining the HA segment from clade 3C.2a1b.2a.2 with the NA segment from clade 3C.2a1b.1a. This reassortment affected 90% (95/106) of H156 subgroup i viruses, including all with T135K substitution, whereas all S156 subgroup iii and iv viruses retained 2a.2 NA. See Supplementary Table S1 for NA characterisation by HA subgroup.

## Epidemiological findings

Participants 1–19 years-old contributed disproportionately to cases (46%; 216/471) vs controls (32%; 312/980) (p < 0.001) and had the highest influenza A(H3N2) per cent positivity (41%; 216/528) (Table 1). We further explored age-related risk by genetic subgroup, re-categorising unvaccinated participants as ≤ 25 vs > 25 years to differentiate those born since 1997 with potential T135 priming, from those born before 1997 with potential K135 or other priming history, as outlined in Supplementary Table S2. Among 619 test-negative controls, 272 (44%) were ≤ 25 years-old. Relative to controls, individuals ≤ 25-year-olds were disproportionately represented among A(H3N2) cases overall (57%; 226/396; p < 0.001) but this was more pronounced for H156 viruses with K135 (80%; 32/40; p < 0.001) than H156 with T135 (49%; 58/118; p = 0.30), or S156 with T135 (56%; 88/156; p = 0.006). Recognising their lower contribution overall, individuals 26–35 years of age, with potential K135 priming history, were comparably represented among influenza A(H3N2) cases (15%; 58/396) and controls (13%; 81/619; p = 0.48) but contributed none of the H156 cases with K135 (0/40; p=0.09). Similar age patterns were observed with restriction to BC and, acknowledging smaller sample size, for A135 viruses in Ontario.

**Table 1:** Participant profile, influenza A(H3N2) test-positive cases and influenza test-negative controls, 2022/23 influenza vaccine effectiveness evaluation, SPSN, Canada, 1 November 2022–6 January 2023 (n = 1,451)

Characteristics	All participants^a^ (column %)							Proportion influenza vaccinated^b^ (row %)						
	Overall		Influenza A(H3N2) cases		Influenza virus test- negative controls		p value^c^	Overall		p value^c^	Influenza A(H3N2) cases^d^		Influenza virus test- negative controls^d^	
	n	%	n	%	n	%		n	%		n	%	n	%
N (row %)	1,451	100	471	32	980	68	NA	436	30	NA	75	16	361	37
**Age group (years)**														
1–19	528	36	216	46	312	32	< 0.001	106	20	< 0.001	25	12	81	26
20–64	704	49	212	45	492	50		184	26		29	14	155	32
≥ 65	219	15	43	9	176	18		146	67		21	49	125	71
Median (range)	33 (1–92)		23 (1–87)		37 (1–92)		< 0.001	50 (1–92)		< 0.001	39 (1–84)		53 (1–92)	
Interquartile range	11–55		9–40		13–59		NA	22–70		NA	13–66		27–71	
**Sex**														
Female	888	61	278	59	610	62	0.19	286	32	0.022	47	17	239	39
Male	551	38	191	41	360	37		146	27		28	15	118	33
Unknown	12	1	2	0	10	1	NA	4	33	NA	0	0	4	40
**Comorbidity^e^**														
No	1,068	74	365	77	703	72	0.008	280	26	< 0.001	45	12	235	33
Yes	299	21	78	17	221	23		133	44		22	28	111	50
Unknown	84	6	28	6	56	6	NA	23	27	NA	8	29	15	27
**Province**														
Alberta	275	19	77	16	198	20	0.20	89	32	< 0.001	12	16	77	39
British Columbia	293	20	97	21	196	20		115	39		21	22	94	48
Ontario	656	45	213	45	443	45		183	28		33	16	150	34
Quebec	227	16	84	18	143	15		49	22		9	11	40	28
**Weeks of specimen collection, 2022/23**														
44–45	131	9	44	9	87	9	< 0.001	30	23	< 0.001	6	14	24	28
46–47	329	23	153	32	176	18		56	17		21	14	35	20
48–49	407	28	166	35	241	25		111	27		25	15	86	36
50–51	382	26	91	19	291	30		132	35		21	23	111	38
52–1	202	14	17	4	185	19		107	53		2	12	105	57

NA: not applicable; SPSN: Sentinel Practitioner Surveillance Network.

^a^ Presenting within 7 days of influenza-like illness onset defined as acute onset of fever and cough and one other symptom including sore throat, myalgia, arthralgia or prostration. Fever was not a required symptom in adults ≥ 65 years.

^b^ Vaccination status based on patient self-report; defined as receipt of 2022/23 seasonal influenza vaccine at least 2 weeks before symptom onset. Patients vaccinated less than 2 weeks before onset of symptoms or with unknown vaccination status or timing were excluded.

^c^ p values for comparison between cases and controls or for the proportion vaccinated were derived by chi-squared test or Wilcoxon rank-sum test.

^d^ Without regard to time before illness onset, 101 of 497 (20%) cases and 406 of 1,025 (40%) controls were vaccinated (p < 0.001).

^e^ Includes chronic comorbidities that place individuals at higher risk of serious complications from influenza as defined by Canada’s National Advisory Committee on Immunization, including: heart, pulmonary (including asthma), renal, metabolic (such as diabetes), blood, cancer or immunocompromising conditions, conditions that compromise management of respiratory secretions and increase risk of aspiration, or morbid obesity (body mass index ≥ 40) [9].

Unless otherwise specified, values displayed in the columns represent the number of specimens per category and percentages are relative to the total.

Crude and adjusted VE estimates are shown in Table 2. Neither sex nor comorbidity affected estimates (based on investigation provided in Supplementary Table S3) and were not included as covariates. Influenza A(H3N2) VE was 54% (95% confidence interval (CI): 38 to 66) overall. VE was similar against vaccine-like S156 viruses at 53% (95% CI: 25 to 70) and H156 viruses at 50% (95% CI: 24 to 67). Against H156 viruses with T135, the VE was 52% (95% CI: 19 to 71) and against H156 viruses with K135 substitution was 46% (95% CI: −13 to 74), the latter similar at 44% (95% CI: −22 to 75) with restriction to BC where most (47/53; 89%) such variants were detected. 

**Table 2:** Vaccine effectiveness estimates against influenza A(H3N2), overall and variant-specific, SPSN, Canada, 1 November 2022–6 January 2023 (weeks 44–1) (n = 1,451)

	Total	Cases						Controls			Unadjusted VE^a^		Adjusted^b^ VE^a^	
	N	n vac^c^	N				%	n vac^c^	N	%	%	95% CI	%	95% CI
Primary analysis	1,451	75	471				16	361	980	37	68	57 to 75	54	38 to 66
**Age-stratified**														
1–19 years	528	25				216	12	81	312	26	63	39 to 77	47	11 to 69
20–64 years	704	29				212	14	155	492	32	66	47 to 78	58	33 to 73
≥ 65 years	219	21				43	49	125	176	71	61	23 to 80	59	15 to 80
**Variant-specific**														
All S156^d^ viruses	1,164	27			184		15	361	980	37	71	55 to 81	53	25 to 70
All H156^e^ viruses	1,187	38		207			18	361	980	37	61	44 to 74	50	24 to 67
H156 with T135^f^	1,120	22	140				16	361	980	37	68	49 to 80	52	19 to 71
H156 with K135^g^, all SPSN provinces	1,033	13	53				25	361	980	37	44	−6 to 71	46	−13 to 74
H156 with K135^g^, British Columbia only	243	13	47				28	94	196	48	59	17 to 79	44	−22 to 75

CI: confidence interval; ECDC: European Centre for Disease Prevention and Control; VE: vaccine effectiveness; OR: odds ratio; PLR: penalised logistic regression; SPSN: Sentinel Practitioner Surveillance Network; vac: vaccinated; VE: vaccine effectiveness.

^a^ OR compared influenza test positivity between vaccinated and unvaccinated participants using logistic regression, adjusting for confounders as specified. VE was calculated as (1 − OR) × 100%.

^b^ Adjusted for age group (1–19, 20–64, ≥ 65 years), province (Alberta, British Columbia, Ontario, Quebec) and calendar time based on bi-weekly categories (44–45, 46–47, 48–49, 50–51, 52–1). Age stratified estimates not further adjusted for finer age sub-categories.

^c^ Vaccination status based on patient self-report; defined as receipt of 2022/23 seasonal influenza vaccine at least 2 weeks before symptom onset. Patients vaccinated less than 2 weeks before onset of symptoms or with unknown vaccination status or timing were excluded.

^d^ 3C.2a1b.2a.2 viruses that are H156S (B) like the A/Darwin/9/2021 (egg-passaged) and A/Darwin/6/2021 (cell-passaged) vaccine strains (i.e. S156 ECDC subgroups ii-iv) [12] (Supplementary Table S1).

^e^ 3C.2a1b.2a.2 viruses that remain H156 (B) and with E50K (C) (as per H156 ECDC subgroup i).

^f^ 3C.2a1b.2a.2 viruses that remain H156 (B) and with E50K (C) (as per H156 ECDC subgroup i) but excluding viruses bearing K or A substitution at position 135 (i.e. T135 viruses, excluding viruses with T135K or T135A (A)(RBS)(−CHO) substitutions).

^g^ 3C.2a1b.2a.2 viruses that remain H156 (B) and with E50K (C) (as per H156 ECDC subgroup i) plus additional substitutions F79V + T135K (A)(RBS)(−CHO) + I140K (A) + G275D(C).

In sensitivity analyses, VE findings were similar using Firth’s method of penalised logistic regression to address small sample size, as shown in Supplementary Table S4 [23,24], and when excluding SARS-CoV-2 test-positive specimens from influenza test-negative controls as shown in Supplementary Table S3 [25].

## Discussion

During an unusually early influenza A(H3N2) epidemic caused by 2a.2 viruses, the 2022/23 influenza vaccine reduced the risk of medically attended illness by about 50%. This VE estimate is higher than typically reported for influenza A(H3N2), often < 40% [8], and higher than estimated by the Canadian SPSN (36%) [1], Europe (29%) [26] or the US (36%) [27] during the delayed 2021/22 influenza A(H3N2) epidemic, similarly caused by 2a.2 viruses. Shorter time since vaccination and/or better match with the updated vaccine may have improved VE. The extent to which relative pause in influenza virus circulation during the coronavirus disease (COVID-19) pandemic may have affected the current VE estimates, is uncertain.

Effectiveness of the S156 vaccine did not differ overall with S vs H variation at position 156, notwithstanding reassorted NA additionally distinguishing most H156 viruses. Among H156 viruses we identified a subset with an additional T135K substitution for which the VE point estimate was below 50%, but with wide CI that overlapped those of other estimates. Although involving different vaccine and circulating strains, the SPSN has previously reported lower VE against T135K-bearing variants in 2016/17 (ca 20%) and 2017/18 (ca 10%), as have European investigators in 2018/19 (ca 10%) [7,28]. Such K135 variants, however, have not predominated overall and were ultimately outcompeted by T135 viruses.

Although geographic variation is not unexpected, it is unclear why viruses with T135K substitution established a stronger foothold in BC than elsewhere during the 2022/23 season. Among 118 other viruses collected in BC for non-SPSN clinical purposes between 2 November and 8 December 2022, 81 (69%) were also H156 with T135K substitution (GISAID identifiers in Supplementary Table S5) [21]. Conversely, among > 17,000 global sequences in GISAID (identifiers in Supplementary Table S6) with collection dates between 1 January and 30 December 2022, just 14 bore T135K, namely three from Saskatchewan, nine from the US and two from Denmark, the earliest of which was collected 3 August 2022 (in the US) [21]. As in Ontario, a greater subset in Europe and the US was instead T135A. Among unvaccinated SPSN participants, those ≤ 25 years born during T135 predominance (since 1997), comprised most (ca 80%) of the K135 variant cases, with no K135 cases observed among potentially K135-primed adults. Our exploration and interpretation in relation to potential imprinting effects is hypothesis-generating only [29]. More definitive evaluation, including the implications for VE estimation, requires larger datasets, similarly standardised for testing indication and vaccine status and conducted across the full epidemic to address potential differences in the timing of age-related transmission and risk [30,31].

We report reassortment whereby H156 clade 3C.2a1b.2a.2 viruses, including those with T135K, acquired NA from clade 3C.2a1b.1a. When that occurred is unknown, with analysis of internal gene segments ongoing, but evidence of the same reassortment this season is found among NA phylogenies available in Nextstrain [14]. Inter-clade reassortments involving NA and other gene segments have been shown in other recent seasons, such as between clades 3C.2a2 and 3C.2a1a in 2017/18 [7,11,14,32] and between clades 3C.3a1 and 3C.2a in 2018/19 [14]. The implications of such seasonal reassortments on epidemic severity, as hypothesised for the 2017/18 season [32], or for VE estimates (noting NA inclusion in vaccines), remains uncertain. Strategic use of WGS in sentinel networks may help further elucidate the prevalence, persistence and potential impact of reassortment viruses.

Our study has limitations, including small sample size with wide CI in stratified analyses. Residual bias and confounding cannot be ruled out. Measuring VE while both vaccine coverage and epidemic risk are evolving makes calendar time adjustment especially challenging but important. We genetically characterised most (> 80%) of our contributing viruses but generalisation elsewhere with a different mix of variants (or vaccines) requires caution.

## Conclusions

During an unusually early influenza A(H3N2) epidemic in Canada, the 2022/23 influenza vaccine provided substantial protection, reducing the risk of medically attended influenza A(H3N2) illness by about half among vaccinated compared with unvaccinated individuals. The potential impact of unique genetic diversity, including T135K substitution and reassortment, warrants further evaluation elsewhere to inform vaccine update and to refine or refute imprinting considerations. 

## Supplementary Data

1151000 Supplement
