# Supplementary material for: Vaccine effectiveness estimates from an early-season influenza A(H3N2) epidemic, including unique genetic diversity with reassortment, Canada, 2022/23
Source: Euro Surveill. 2023 Feb 2;28(5):2300043. doi: 10.2807/1560-7917.ES.2023.28.5.2300043 (PMC9896608; doi:10.2807/1560-7917.ES.2023.28.5.2300043)
Supplement: Supplement [file 23-00043_SKOWRONSKI_Supplement.pdf]

Supplementary Materials

This supplementary material is hosted by *Eurosurveillance* as supporting information alongside the article "*Vaccine effectiveness estimates from an early-season influenza A(H3N2) epidemic, including unique genetic diversity with reassortment, Canada, 2022/23*" on behalf of the authors who remain responsible for the accuracy and appropriateness of the content. The same standards for ethics, copyright, attributions and permissions as for the article apply. Supplements are not edited by *Eurosurveillance* and the journal is not responsible for the maintenance of any links or email addresses provided therein.

Table of Contents [hyperlinked]

Supplementary Table S1. Influenza A(H3N2) clade 3C.2a1b.2a.2 cases by genetic subgroup included in 2022/23 influenza vaccine effectiveness analyses of the Canadian Sentinel Practitioner Surveillance Network (SPSN)(n=391).. **3**

Supplementary Table S2. Distribution of influenza test-negative controls and A(H3N2) cases (unvaccinated SPSN participants) by genetic subgroup, and age categories re-defined by potential priming history at amino acid position 135 of the hemagglutinin (≤ 25 versus >25 years of age) **4**

Supplementary Table S3. Comparison of primary 2022/23 influenza A(H3N2) vaccine effectiveness estimates of the SPSN, unadjusted and with univariate and full covariate adjustment, with/without sex and comorbidity and with/without exclusion of SARS-CoV-2 test-positive specimens from influenza test-negative controls.. **5**

Supplementary Table S4. Vaccine effectiveness estimates against influenza A(H3N2), age-stratified and variant-specific, applying Firth's method of penalized logistic regression, SPSN, Canada, 1 November 2022—6 January 2023 (weeks 44—1) (n=1,451)....................................... **6**

Supplementary Table S5. Hemagglutinin (HA) sequences from A(H3N2) viruses identified in non-SPSN specimens in British Columbia, Canada, 2 November to 8 December 2022 (N=118) ... **7**

Supplementary Table S6. Global hemagglutinin (HA) sequences outside of British Columbia, Canada genetically characterized as 3C.2a1b.2a.2 subgroup i viruses with T135K substitution, 2 November to 28 December 2022 (N=14) **10**

References, Supplementary Material... **11**

Supplementary Table S1. Influenza A(H3N2) clade 3C.2a1b.2a.2 cases by genetic subgroup included in 2022/23 influenza vaccine effectiveness analyses of the Canadian Sentinel Practitioner Surveillance Network (SPSN)(n=391)

Hemagglutinin surface protein, subunit 1 (HA1)[1]							Neuraminidase (NA)[1] N=154	
**3C.2a1b.2a.2 genetic subgroups as defined by ECDC [1] (with corresponding Nextstrain terminology) [2] based on specified HA1 amino acid substitutions[2,3] (antigenic site)**<br>+ select additional substitutions identified among SPSN viruses (antigenic site)	**Amino acid at position 156[4]**	**British Columbia N=89 n/N (%)**	**Alberta N=62 n/N (%)**	**Ontario N=163 n/N (%)**	**Quebec N=77 n/N (%)**	**TOTAL N=391 n/N (%)**	**n/N**	**Clade[5]**
**Genetic subgroup i (Nextstrain "2b") = 3C.2a1b.2a.2 + E50K (C)[6]**	H	**74 (83%)**	**40 (65%)**	**53 (33%)**	**40 (52%)**	**207 (53%)**		
+ F79V + I140K (A)		4	7	27	23[7]	61	17/17	3C.2a1b.1a
+ F79V + I140K (A) + R33Q + S262N (E)		6	6	1	1	14	11/11	3C.2a1b.2a.2
+ F79V + I140K (A) + I242M (D)		0	0	5[8]	14	19	10/10	
+ F79I + I140K (A) + I242M (D)		16	21	1	1	39	26/26	3C.2a1b.1a
+ F79I + I140K (A) + I242M (D) + S145N (A)[9]		1	3	3	0	7	2/2	
+ F79V + I140K (A) + **T135K (A)(RBS)(-CHO)[10]** + G275D (C)		47	3	2	1	53	40/40	3C.2a1b.1a
+ F79V + I140K (A) + **T135A (A)(RBS)(-CHO)[10]** + S262N (E)		0	0	14	0	14	—	—
**Genetic subgroup ii (Nextstrain "2a.3") = 3C.2a1b.2a.2 + D53N (C) + N96S (D)(+CHO) + H156S (B)[4] + I192F (B)**	S	**0**	**0**	**3 (2%)**	**0**	**3 (<1%)**	—	—
+ E50K (C) + I140K (A) + I223V (Nextstrain "2a.3a.1")		0	0	3	0	3		
**Genetic subgroup iii (Nextstrain "2a.1") = 3C.2a1b.2a.2 + D53G (C) + H156S (B)[4,11]**	S	**5 (6%)**	**2 (3%)**	**3 (2%)**	**0**	**10 (3%)**	5/5	3C.2a1b.2a.2
+ D104G + K276R (C)		5	2	3	0	10		
**Genetic subgroup iv (Nextstrain "2a.1b") = 3C.2a1b.2a.2 + D53G (C) + D104G + I140K (A) + H156S (B)[4] + K276R (C) + R299K (C)**	S	**10 (11%)**	**20 (32%)**	**104[12] (64%)**	**37 (48%)**	**171 (44%)**	43/43	3C.2a1b.2a.2
+ **T135K (A)(RBS)(-CHO)[10]**		**1**	**1**	**0**	**0**	**2**		

-/+CHO = loss or gain of potential glycosylation site; ECDC = European Centre for Disease Control and Prevention; RBS = receptor binding site (adjacent)

[1] HA1 based on Sanger and/or whole genome sequencing (WGS); neuraminidase based on WGS. WGS applied to only a subset of viruses (N=154) distributed as shown.
[2] Substitutions relative to A/Bangladesh/4005/2020, 3C.2a1b.2a2 reference virus with HA1 substitutions Y159N (B), T160I (B)(-CHO), L164Q (B), G186D (B), D190N (B)(RBS), F193S (B), Y195F (RBS) [1]
[3] The cell-passaged A/Darwin/6/2021 and the egg-passaged A/Darwin/9/2021 vaccine strains recommended for the 2022-23 northern hemisphere influenza season (and the 2022 and 2023 southern hemisphere influenza seasons) are H156S (B) relative to A/Bangladesh/4005/2020, i.e. they are S156. The cell-passaged version is also D53G (C) and belongs within ECDC subgroup iii (without the other commonly cited substitutions). However, the egg-passaged A/Darwin/9/2021 vaccine strain does not cluster within the specified ECDC subgroups of circulating viruses, and additionally acquired recognized egg-adaptation substitutions, D186N (B) plus D225G [1]
[4] **Amino acid position 156 is a major antigenic cluster transition site** as specified in Supplementary References [3]
[5] Grey shading signifies reassorted neuraminidase.
[6] ECDC defines subgroup i as E50K (C) with a range of additional substitutions, e.g. F79V, I140K (A), S262N (E) and R33Q [1]. Select substitutions identified among SPSN viruses are displayed here.
[7] Includes one virus with the reversion K50E (C).
[8] Includes one virus with drifted K50I (C).
[9] **Amino acid position 145 is a major antigenic cluster transition site** as specified in Supplementary References [3].
[10] **Amino acid position 135 is a major antigenic cluster transition accessory site** as specified in Supplementary References [3].
[11] ECDC defines subgroup iii as D53G, H156S (B), commonly with D104G and K276R (C), and further subdivided into clusters defined by either T135A (A)(RBS)(-CHO) + T167S (D) or E50K (C) + R299K (C) [1]. Additional clusters were not observed within SPSN subgroup iii viruses.
[12] Includes one virus with drifted G104S and one virus with drifted R276S (C).

Supplementary Table S2. Distribution of influenza test-negative controls and A(H3N2) cases (unvaccinated SPSN participants) by genetic subgroup, and age categories re-defined by potential priming history at amino acid position 135 of the hemagglutinin (≤ 25 versus >25 years of age)

Age in 2022 (years)[1]	Birth years	Potential A(H3N2) priming exposure at HA1 position 135[2]	Influenza test-negative controls N=619 n (%)	Influenza A(H3N2) cases by clade 3C.2a1b.2a.2 genetic subgroups [as per [1] and Supplementary Table 1]					
				All A(H3N2) viruses N=396 n (%)	Genetically characterized A(H3N2) viruses N=326 n (%)	S156 and T135[3] N=156[4] n (%)	H156 and T135[5] N=118 n (%)	H156 and K135[6,7] N=40 n (%)	H156 and A135[8,9] N=11 n
1-25 years	1997 to current	T	272 (44)	226 (57)	187 (57)	88 (56)	58 (49)	32 (80)	9
>25 years	1996 and earlier	T, K, E, or G	347 (56)	170 (43)	139 (43)	68 (44)	60 (51)	8 (20)	2
26-29	1993 to 1996	T or K	28 (5)	19 (5)	17 (5)	10 (6)	7 (6)	0	0
30-35	1987 to 1992	K, E or G	53 (9)	39 (10)	34 (10)	21 (13)	13 (11)	0	0
36-60	1962-1986	G	183 (30)	79 (20)	61 (19)	25 (16)[4]	27 (23)	7 (18)	1
≥61	1961 and earlier	G	83 (13)	33 (8)	27 (8)	12 (8)	13 (11)	1 (3)	1

HA1 = hemagglutinin surface protein, subunit 1
A = alanine; E = glutamic acid; G = glycine; H = histidine; K = lysine; T = threonine

---

[1] Age groups predicated on amino acid exposure before/since emergence of A/Sydney/5/97 A(H3N2) in 1997. During the period 1993-1996, K135 viruses briefly predominated but emergence of A/Sydney/5/97 was accompanied by **K135T (A)(RBS)(+CHO)** substitution after which T135 predominated among circulating A(H3N2) strains [3]. Age category ≤ 25 years does not take into account A(H3N2) clade 3C.2a1a viruses in 2016-17 or 3C.2a1b.1 viruses in 2017-18 also possessing **T135K (A)(RBS)(-CHO)** substitution, potentially relevant to young children. However, these variants did not predominate and by 2021-22 were outcompeted by 3C.2a1b.2 viruses that were **T135 (A)(RBS)(+CHO)**. See Supplementary References [2,5,6].

[2] Based on first childhood exposure to specified amino acid identified historically among circulating A(H3N2) viruses since their emergence during the 1968 pandemic, recognizing it may take several years for the first childhood priming exposure to occur. See Supplementary References [6-7]. Does not consider other priming history such as to H1 or H2 subtypes that may have also played a role.

[3] 3C.2a1b.2a.2 viruses that are **H156S (B)** like the A/Darwin/9/2021 (egg-passaged) and A/Darwin/6/2021 (cell-passaged) vaccine strains (i.e. S156 ECDC subgroups ii-iv) [1] (**Supplementary Table 1**)

[4] One ECDC subgroup iv virus from British Columbia that is S156, K135 excluded from these analyses.

[5] 3C.2a1b.2a.2 viruses that remain **H156 (B)** and with **E50K(C)** (as per ECDC subgroup i) but excluding viruses bearing K or A substitution at position 135 (i.e. T135 viruses, excluding viruses with T135K or T135A (A)(RBS)(-CHO) substitutions [1] (**Supplementary Table 1**).

[6] 3C.2a1b.2a.2 viruses that remain **H156 (B)** and with **E50K (C)** (as per ECDC subgroup i) plus additional substitutions **F79V + T135K (A)(RBS)(-CHO) + I140K (A) + G275D (C)** [1] (**Supplementary Table 1**).

[7] Of H156 viruses that were K135 among unvaccinated participants, 34/40 (85%) were identified in British Columbia (BC), including 29/32 (91%) cases aged ≤ 25 years and 5/8 (63%) cases aged >25 years (all 36-60 years). Compared to test-negative controls of which 57/102 (56%) were ≤ 25 years, 49/76 (64%) A(H3N2) cases overall (p=0.25), 44/69 (64%) genetically characterized A(H3N2) cases (p=0.30), 5/11 (45%) cases with viruses that were S156 and T135 (p=0.51), 10/23 (43%) cases with viruses that were H156 and T135 (p=0.28) and 29/34 (85%) cases with viruses that were H156 and K135 (p=0.004) were aged ≤ 25 years in BC (unvaccinated participants).

[8] 3C.2a1b.2a.2 viruses that remain **H156 (B)** and with **E50K (C)** (as per ECDC subgroup i) plus additional substitutions **F79V + T135A (A)(RBS)(-CHO) + I140K (A) + S262N (E)** [1] (**Supplementary Table 1**).

[9] Of H156 viruses that were A135, 11/11 among unvaccinated participants were identified in Ontario. Compared to influenza test-negative controls of which 146/293 (50%) were ≤ 25 years, 107/180 (59%) A(H3N2) cases overall (p=0.04), 85/136 (63%) genetically characterized A(H3N2) cases (p=0.01), 57/92 (62%) cases with viruses that were S156 and T135 (p=0.04), 19/31 (61%) cases with viruses that were H156 and T135 (p=0.23) and 9/11 (82%) cases with viruses that were H156 and A135 (p=0.06) were aged ≤ 25 years in Ontario (unvaccinated participants).

Supplementary Table S3. Comparison of primary 2022/23 influenza A(H3N2) vaccine effectiveness estimates of the SPSN, unadjusted and with univariate and full covariate adjustment, with/without sex and comorbidity and with/without exclusion of SARS-CoV-2 test-positive specimens from influenza test-negative controls

	**Including COVID-19 test-positive cases among influenza test-negative controls**		**Excluding SARS-CoV-2 test-positive cases from influenza test-negative controls[1]**	
**Adjustment including for sex and comorbidity[2]**				
**Sample size**	**n vacc[3]/N**	**%**	**n vac/N**	**%**
Total	1,359		1,253	
Cases	67/441	15	67/441	15
Controls	343/918	37	300/812	37
**Vaccine effectiveness[4]**	**%**	**95% CI**	**%**	**95% CI**
Unadjusted	70	(60, 78)	69	(59, 77)
**Univariate adjustment for:**				
- Age group (1–19, 20–64, ≥ 65 years)	67	(55, 75)	67	(54, 75)
- Province (AB, BC, ON, QC)	70	(60, 78)	70	(59, 78)
- Bi-weekly (44-45, 46-47, 48-49, 50-51, 52-1)	62	(48, 72)	61	(47, 72)
- Sex	70	(60, 77)	69	(59, 77)
- Comorbidity[5]	69	(59, 77)	69	(58, 77)
Full covariate adjustment[6]	57	(41, 69)	58	(42, 70)
**Adjustment, not including sex and comorbidity (as per primary analysis)[7]**				
**Sample size**	**n vacc/N**	**%**	**n vac/N**	**%**
Total	1,451		1,339	
Cases	75/471	16	75/471	16
Controls	361/980	37	317/868	37
**Vaccine effectiveness**	**%**	**95% CI**	**%**	**95% CI**
Unadjusted	68	(57, 75)	67	(56, 75)
**Univariate adjustment for:**				
- Age group (1–19, 20–64, ≥ 65 years)	64	(51, 73)	64	(52, 73)
- Province (AB, BC, ON, QC)	68	(57, 76)	67	(57, 76)
- Bi-weekly (44-45, 46-47, 48-49, 50-51, 52-1)	60	(46, 70)	59	(45, 70)
Full covariate adjustment[8]	54	(38, 66)	55	(39, 67)

AB = Alberta; BC = British Columbia; ON = Ontario; QC = Quebec; CI: confidence interval; VE: vaccine effectiveness; vacc=vaccinated

[1] Sensitivity analysis in which Severe Acute Respiratory Syndrome Coronavirus 2 (SARS-CoV-2) [the cause of the Coronavirus Disease 2019 (COVID-19 pandemic) test-positive specimens are excluded from influenza test-negative controls to assess potential bias associated with correlation between COVID-19 and influenza vaccination as per Doll et al [8]. Note that co-infections between influenza A(H3N2) cases and SARS-CoV-2 are retained.

[2] Sensitivity analyses in which adjustment includes sex and comorbidity as covariates and individuals with missing sex and comorbidity information are excluded as per other covariates with missing information

[3] Vaccination status based on patients' self-report; defined as receipt of 2022/23 seasonal influenza vaccine at least 2 weeks before symptom onset. Patients vaccinated less than 2 weeks before onset of symptoms or with unknown vaccination status or timing were excluded.

[4] Odds ratios (ORs) compared influenza test positivity between vaccinated and unvaccinated participants using logistic regression, adjusting for confounders as specified. VE was calculated as (1 − OR) × 100%.

[5] Includes chronic comorbidities that place individuals at higher risk of serious complications from influenza as defined by Canada's National Advisory Committee on Immunization, including: heart, pulmonary (including asthma), renal, metabolic (such as diabetes), blood, cancer or immunocompromising conditions, conditions that compromise management of respiratory secretions and increase risk of aspiration, or morbid obesity (body mass index ≥ 40).

[6] Age group (1–19, 20–64, ≥ 65 years), sex (male, female), comorbidity (yes, no), province (AB, BC, ON, QC), and calendar time based on bi-week categories (44-45, 46-47, 48-49, 50-51,52-1). Individuals with missing sex and comorbidity information additionally excluded.

[7] All analyses include individuals with missing sex and comorbidity information as these are no longer included in covariate adjustment

[8] Age group (1–19, 20–64, ≥ 65 years), province (AB, BC, ON, QC), and calendar time based on bi-week categories (44-45, 46-47, 48-49, 50-51,52-1)

Supplementary Table S4. Vaccine effectiveness estimates against influenza A(H3N2), age-stratified and variant-specific, applying Firth's method of penalized logistic regression, SPSN, Canada, 1 November 2022—6 January 2023 (weeks 44—1) (n=1,451)

	Total	Cases			Controls			Unadjusted VE[a]		Adjusted[b] VE[a]	
	N	n vac[c]	N	%	n vac[c]	N	%	%	95% CI	%	95% CI
Primary analysis	1,451	75	471	16	361	980	37	67	57 to 75	54	38 to 66
**Age-stratified**											
1–19 years	528	25	216	12	81	312	26	62	39 to 77	46	11 to 69
20–64 years	704	29	212	14	155	492	32	65	47 to 78	57	33 to 73
≥ 65 years	219	21	43	49	125	176	71	61	23 to 80	57	13 to 79
**Variant-specific**											
All S156[d] viruses	1,164	27	184	15	361	980	37	70	55 to 81	52	25 to 70
All H156[e] viruses	1,187	38	207	18	361	980	37	61	44 to 74	49	24 to 67
H156 with T135[f]	1,120	22	140	16	361	980	37	67	49 to 80	51	19 to 71
H156 with K135[g], all SPSN provinces	1,033	13	53	25	361	980	37	43	−5 to 71	44	−13 to 74
H156 with K135[g], British Columbia only	243	13	47	28	94	196	48	58	17 to 79	43	−21 to 74

CI: confidence interval; ECDC: European Centre for Disease Prevention and Control; VE: vaccine effectiveness; OR: odds ratio; SPSN: Sentinel Practitioner Surveillance Network; vac: vaccinated; VE: vaccine effectiveness.

[a] OR compared influenza test positivity between vaccinated and unvaccinated participants using logistic regression, adjusting for confounders as specified. VE was calculated as (1 − OR) × 100%.

[b] Adjusted for age group (1–19, 20–64, ≥ 65 years), province (Alberta, British Columbia, Ontario, Quebec) and calendar time based on bi-weekly categories (44–45, 46–47, 48–49, 50–51, 52–1). Age stratified estimates not further adjusted for finer age sub-categories.

[c] Vaccination status based on patient self-report; defined as receipt of 2022/23 seasonal influenza vaccine at least 2 weeks before symptom onset. Patients vaccinated less than 2 weeks before onset of symptoms or with unknown vaccination status or timing were excluded.

[d] 3C.2a1b.2a.2 viruses that are H156S (B) like the A/Darwin/9/2021 (egg-passaged) and A/Darwin/6/2021 (cell-passaged) vaccine strains (i.e. S156 ECDC subgroups ii-iv) (Supplementary Table S1) [1]

[e] 3C.2a1b.2a.2 viruses that remain H156 (B) and with E50K (C) (as per H156 ECDC subgroup i).

[f] 3C.2a1b.2a.2 viruses that remain H156 (B) and with E50K (C) (as per H156 ECDC subgroup i) but excluding viruses bearing K or A substitution at position 135 (i.e. T135 viruses, excluding viruses with T135K or T135A (A)(RBS)(−CHO) substitutions).

[g] 3C.2a1b.2a.2 viruses that remain H156 (B) and with E50K (C) (as per H156 ECDC subgroup i) plus additional substitutions F79V + T135K (A)(RBS)(−CHO) + I140K (A) + G275D(C).

Supplementary Table S5. Hemagglutinin (HA) sequences from A(H3N2) viruses identified in non-SPSN specimens in British Columbia, Canada, 2 November to 8 December 2022 (N=118)*

*Includes clinical specimens collected in British Columbia, Canada that were not collected as part of the Canadian Sentinel Practitioner Surveillance Network (SPSN). Sequences obtained from the EpiFlu database of the Global Initiative on Sharing All Influenza Data (GISAID) [4] available up to January 10, 2023, with collection dates spanning 2 November to 8 December 2022 (n=118).

All sequences shown below in **Supplementary Table 6** have originating and submitting lab of the British Columbia Centre for Disease Control, with authors Prystajecky,Natalie; Tyson,John; Jassem,Agatha; Kuchinski,Kevin; Lee,Tracy; Azana,Rob; Fung,Janet; Chan,Michael; Cheung,Branco; Caleta,Jessica; Tsang,Frankie; Russell,Shannon; Zlosnik,James; Hoang,Linda

Segment ID	Segment	Country	Collection Date (yyyy-mm-dd)	Isolate ID	Isolate Name
EPI2282109	HA	Canada	2022-Dec-08	EPI_ISL_16401620	A/British Columbia/PHL-503/2022
EPI2282093	HA	Canada	2022-Dec-07	EPI_ISL_16401617	A/British Columbia/PHL-470/2022
EPI2282082	HA	Canada	2022-Dec-06	EPI_ISL_16401615	A/British Columbia/PHL-493/2022
EPI2282072	HA	Canada	2022-Dec-03	EPI_ISL_16401613	A/British Columbia/PHL-472/2022
EPI2282060	HA	Canada	2022-Dec-05	EPI_ISL_16401611	A/British Columbia/PHL-481/2022
EPI2282055	HA	Canada	2022-Dec-04	EPI_ISL_16401610	A/British Columbia/PHL-440/2022
EPI2282037	HA	Canada	2022-Dec-03	EPI_ISL_16401606	A/British Columbia/PHL-448/2022
EPI2282033	HA	Canada	2022-Dec-01	EPI_ISL_16401605	A/British Columbia/PHL-492/2022
EPI2282023	HA	Canada	2022-Dec-01	EPI_ISL_16401603	A/British Columbia/PHL-459/2022
EPI2282012	HA	Canada	2022-Dec-01	EPI_ISL_16401601	A/British Columbia/PHL-480/2022
EPI2282008	HA	Canada	2022-Dec-01	EPI_ISL_16401600	A/British Columbia/PHL-482/2022
EPI2282003	HA	Canada	2022-Dec-01	EPI_ISL_16401599	A/British Columbia/PHL-501/2022
EPI2262689	HA	Canada	2022-Nov-18	EPI_ISL_16283463	A/British Columbia/PHL-257/2022
EPI2262681	HA	Canada	2022-Nov-17	EPI_ISL_16283462	A/British Columbia/PHL-269/2022
EPI2262673	HA	Canada	2022-Nov-17	EPI_ISL_16283461	A/British Columbia/PHL-281/2022
EPI2262665	HA	Canada	2022-Nov-16	EPI_ISL_16283460	A/British Columbia/PHL-293/2022
EPI2262657	HA	Canada	2022-Nov-16	EPI_ISL_16283459	A/British Columbia/PHL-305/2022
EPI2262649	HA	Canada	2022-Nov-16	EPI_ISL_16283458	A/British Columbia/PHL-317/2022
EPI2262641	HA	Canada	2022-Nov-15	EPI_ISL_16283457	A/British Columbia/PHL-328/2022
EPI2262636	HA	Canada	2022-Nov-15	EPI_ISL_16283456	A/British Columbia/PHL-246/2022
EPI2262628	HA	Canada	2022-Nov-13	EPI_ISL_16283455	A/British Columbia/PHL-258/2022
EPI2262620	HA	Canada	2022-Nov-13	EPI_ISL_16283454	A/British Columbia/PHL-270/2022
EPI2262607	HA	Canada	2022-Nov-30	EPI_ISL_16283452	A/British Columbia/PHL-364/2022
EPI2262601	HA	Canada	2022-Nov-30	EPI_ISL_16283451	A/British Columbia/PHL-386/2022
EPI2262578	HA	Canada	2022-Nov-28	EPI_ISL_16283448	A/British Columbia/PHL-375/2022
EPI2262571	HA	Canada	2022-Nov-28	EPI_ISL_16283447	A/British Columbia/PHL-363/2022
EPI2262534	HA	Canada	2022-Nov-25	EPI_ISL_16283442	A/British Columbia/PHL-352/2022
EPI2262526	HA	Canada	2022-Nov-24	EPI_ISL_16283441	A/British Columbia/PHL-403/2022
EPI2262518	HA	Canada	2022-Nov-22	EPI_ISL_16283440	A/British Columbia/PHL-324/2022
EPI2262510	HA	Canada	2022-Nov-20	EPI_ISL_16283439	A/British Columbia/PHL-312/2022
EPI2262502	HA	Canada	2022-Nov-19	EPI_ISL_16283438	A/British Columbia/PHL-262/2022
EPI2262494	HA	Canada	2022-Nov-19	EPI_ISL_16283437	A/British Columbia/PHL-283/2022
EPI2262486	HA	Canada	2022-Nov-19	EPI_ISL_16283436	A/British Columbia/PHL-310/2022
EPI2262476	HA	Canada	2022-Nov-19	EPI_ISL_16283434	A/British Columbia/PHL-251/2022
EPI2262469	HA	Canada	2022-Nov-19	EPI_ISL_16283433	A/British Columbia/PHL-311/2022

Segment ID	Segment	Country	Collection Date (yyyy-mm-dd)	Isolate ID	Isolate Name
EPI2262461	HA	Canada	2022-Nov-19	EPI_ISL_16283432	A/British Columbia/PHL-275/2022
EPI2262453	HA	Canada	2022-Nov-19	EPI_ISL_16283431	A/British Columbia/PHL-271/2022
EPI2262415	HA	Canada	2022-Nov-02	EPI_ISL_16283424	A/British Columbia/PHL-249/2022
EPI2262392	HA	Canada	2022-Nov-08	EPI_ISL_16283421	A/British Columbia/PHL-296/2022
EPI2262374	HA	Canada	2022-Nov-08	EPI_ISL_16283418	A/British Columbia/PHL-318/2022
EPI2262366	HA	Canada	2022-Nov-08	EPI_ISL_16283417	A/British Columbia/PHL-308/2022
EPI2262355	HA	Canada	2022-Nov-08	EPI_ISL_16283415	A/British Columbia/PHL-261/2022
EPI2262339	HA	Canada	2022-Nov-06	EPI_ISL_16283413	A/British Columbia/PHL-329/2022
EPI2262331	HA	Canada	2022-Nov-07	EPI_ISL_16283412	A/British Columbia/PHL-331/2022
EPI2262323	HA	Canada	2022-Nov-05	EPI_ISL_16283411	A/British Columbia/PHL-273/2022
EPI2262315	HA	Canada	2022-Nov-07	EPI_ISL_16283410	A/British Columbia/PHL-320/2022
EPI2262308	HA	Canada	2022-Nov-08	EPI_ISL_16283409	A/British Columbia/PHL-272/2022
EPI2262293	HA	Canada	2022-Nov-07	EPI_ISL_16283407	A/British Columbia/PHL-244/2022
EPI2262271	HA	Canada	2022-Nov-05	EPI_ISL_16283404	A/British Columbia/PHL-216/2022
EPI2262258	HA	Canada	2022-Nov-06	EPI_ISL_16283402	A/British Columbia/PHL-236/2022
EPI2262242	HA	Canada	2022-Nov-07	EPI_ISL_16283400	A/British Columbia/PHL-180/2022
EPI2262226	HA	Canada	2022-Nov-07	EPI_ISL_16283398	A/British Columbia/PHL-198/2022
EPI2262219	HA	Canada	2022-Nov-07	EPI_ISL_16283397	A/British Columbia/PHL-206/2022
EPI2262211	HA	Canada	2022-Nov-05	EPI_ISL_16283396	A/British Columbia/PHL-222/2022
EPI2262204	HA	Canada	2022-Nov-07	EPI_ISL_16283395	A/British Columbia/PHL-217/2022
EPI2262196	HA	Canada	2022-Nov-06	EPI_ISL_16283394	A/British Columbia/PHL-227/2022
EPI2262191	HA	Canada	2022-Nov-06	EPI_ISL_16283393	A/British Columbia/PHL-265/2022
EPI2262175	HA	Canada	2022-Nov-06	EPI_ISL_16283391	A/British Columbia/PHL-172/2022
EPI2262167	HA	Canada	2022-Nov-06	EPI_ISL_16283390	A/British Columbia/PHL-203/2022
EPI2262145	HA	Canada	2022-Nov-02	EPI_ISL_16283387	A/British Columbia/PHL-330/2022
EPI2262132	HA	Canada	2022-Nov-04	EPI_ISL_16283385	A/British Columbia/PHL-177/2022
EPI2262124	HA	Canada	2022-Nov-06	EPI_ISL_16283384	A/British Columbia/PHL-234/2022
EPI2262117	HA	Canada	2022-Nov-04	EPI_ISL_16283383	A/British Columbia/PHL-242/2022
EPI2262109	HA	Canada	2022-Nov-04	EPI_ISL_16283382	A/British Columbia/PHL-232/2022
EPI2262093	HA	Canada	2022-Nov-05	EPI_ISL_16283380	A/British Columbia/PHL-207/2022
EPI2262085	HA	Canada	2022-Nov-06	EPI_ISL_16283379	A/British Columbia/PHL-211/2022
EPI2262078	HA	Canada	2022-Nov-05	EPI_ISL_16283378	A/British Columbia/PHL-223/2022
EPI2262070	HA	Canada	2022-Nov-06	EPI_ISL_16283377	A/British Columbia/PHL-228/2022
EPI2262062	HA	Canada	2022-Nov-05	EPI_ISL_16283376	A/British Columbia/PHL-194/2022
EPI2262054	HA	Canada	2022-Nov-06	EPI_ISL_16283375	A/British Columbia/PHL-238/2022
EPI2262046	HA	Canada	2022-Nov-06	EPI_ISL_16283374	A/British Columbia/PHL-184/2022
EPI2262030	HA	Canada	2022-Nov-06	EPI_ISL_16283372	A/British Columbia/PHL-204/2022
EPI2262023	HA	Canada	2022-Nov-05	EPI_ISL_16283371	A/British Columbia/PHL-176/2022
EPI2262015	HA	Canada	2022-Nov-05	EPI_ISL_16283370	A/British Columbia/PHL-205/2022
EPI2262009	HA	Canada	2022-Nov-05	EPI_ISL_16283369	A/British Columbia/PHL-263/2022
EPI2262001	HA	Canada	2022-Nov-05	EPI_ISL_16283368	A/British Columbia/PHL-241/2022
EPI2261993	HA	Canada	2022-Nov-05	EPI_ISL_16283367	A/British Columbia/PHL-299/2022
EPI2261985	HA	Canada	2022-Nov-05	EPI_ISL_16283366	A/British Columbia/PHL-323/2022
EPI2261977	HA	Canada	2022-Nov-03	EPI_ISL_16283365	A/British Columbia/PHL-287/2022
EPI2261969	HA	Canada	2022-Nov-05	EPI_ISL_16283364	A/British Columbia/PHL-235/2022

Segment ID	Segment	Country	Collection Date (yyyy-mm-dd)	Isolate ID	Isolate Name
EPI2261961	HA	Canada	2022-Nov-04	EPI_ISL_16283363	A/British Columbia/PHL-288/2022
EPI2261954	HA	Canada	2022-Nov-05	EPI_ISL_16283362	A/British Columbia/PHL-182/2022
EPI2261938	HA	Canada	2022-Nov-05	EPI_ISL_16283360	A/British Columbia/PHL-231/2022
EPI2261930	HA	Canada	2022-Nov-06	EPI_ISL_16283359	A/British Columbia/PHL-192/2022
EPI2261922	HA	Canada	2022-Nov-04	EPI_ISL_16283358	A/British Columbia/PHL-220/2022
EPI2261914	HA	Canada	2022-Nov-05	EPI_ISL_16283357	A/British Columbia/PHL-201/2022
EPI2261909	HA	Canada	2022-Nov-05	EPI_ISL_16283356	A/British Columbia/PHL-179/2022
EPI2261901	HA	Canada	2022-Nov-05	EPI_ISL_16283355	A/British Columbia/PHL-188/2022
EPI2261893	HA	Canada	2022-Nov-04	EPI_ISL_16283354	A/British Columbia/PHL-334/2022
EPI2261880	HA	Canada	2022-Nov-02	EPI_ISL_16283352	A/British Columbia/PHL-219/2022
EPI2261864	HA	Canada	2022-Nov-06	EPI_ISL_16283350	A/British Columbia/PHL-230/2022
EPI2261856	HA	Canada	2022-Nov-06	EPI_ISL_16283349	A/British Columbia/PHL-193/2022
EPI2261848	HA	Canada	2022-Nov-06	EPI_ISL_16283348	A/British Columbia/PHL-240/2022
EPI2261840	HA	Canada	2022-Nov-06	EPI_ISL_16283347	A/British Columbia/PHL-183/2022
EPI2261832	HA	Canada	2022-Nov-06	EPI_ISL_16283346	A/British Columbia/PHL-175/2022
EPI2261816	HA	Canada	2022-Nov-06	EPI_ISL_16283344	A/British Columbia/PHL-285/2022
EPI2261803	HA	Canada	2022-Nov-08	EPI_ISL_16283342	A/British Columbia/PHL-264/2022
EPI2261795	HA	Canada	2022-Nov-06	EPI_ISL_16283341	A/British Columbia/PHL-309/2022
EPI2261787	HA	Canada	2022-Nov-05	EPI_ISL_16283340	A/British Columbia/PHL-321/2022
EPI2261772	HA	Canada	2022-Nov-03	EPI_ISL_16283338	A/British Columbia/PHL-306/2022
EPI2261764	HA	Canada	2022-Nov-08	EPI_ISL_16283337	A/British Columbia/PHL-274/2022
EPI2261756	HA	Canada	2022-Nov-02	EPI_ISL_16283336	A/British Columbia/PHL-307/2022
EPI2261748	HA	Canada	2022-Nov-06	EPI_ISL_16283335	A/British Columbia/PHL-187/2022
EPI2261740	HA	Canada	2022-Nov-06	EPI_ISL_16283334	A/British Columbia/PHL-196/2022
EPI2261732	HA	Canada	2022-Nov-05	EPI_ISL_16283333	A/British Columbia/PHL-224/2022
EPI2261727	HA	Canada	2022-Nov-05	EPI_ISL_16283332	A/British Columbia/PHL-247/2022
EPI2261711	HA	Canada	2022-Nov-03	EPI_ISL_16283330	A/British Columbia/PHL-245/2022
EPI2261703	HA	Canada	2022-Nov-04	EPI_ISL_16283329	A/British Columbia/PHL-191/2022
EPI2261695	HA	Canada	2022-Nov-06	EPI_ISL_16283328	A/British Columbia/PHL-276/2022
EPI2261690	HA	Canada	2022-Nov-05	EPI_ISL_16283327	A/British Columbia/PHL-322/2022
EPI2261682	HA	Canada	2022-Nov-05	EPI_ISL_16283326	A/British Columbia/PHL-208/2022
EPI2261674	HA	Canada	2022-Nov-03	EPI_ISL_16283325	A/British Columbia/PHL-389/2022
EPI2261672	HA	Canada	2022-Nov-03	EPI_ISL_16283324	A/British Columbia/PHL-295/2022
EPI2261661	HA	Canada	2022-Nov-02	EPI_ISL_16283322	A/British Columbia/PHL-335/2022
EPI2261655	HA	Canada	2022-Nov-03	EPI_ISL_16283321	A/British Columbia/PHL-351/2022
EPI2261648	HA	Canada	2022-Nov-03	EPI_ISL_16283320	A/British Columbia/PHL-186/2022
EPI2261632	HA	Canada	2022-Nov-03	EPI_ISL_16283318	A/British Columbia/PHL-252/2022
EPI2261602	HA	Canada	2022-Nov-02	EPI_ISL_16283314	A/British Columbia/PHL-297/2022

Supplementary Table S6. Global hemagglutinin (HA) sequences outside of British Columbia, Canada genetically characterized as 3C.2a1b.2a.2 subgroup i viruses with T135K substitution, 2 November to 28 December 2022 (N=14)*

*Sequences obtained from the EpiFlu database of the Global Initiative on Sharing All Influenza Data (GISAID) [4] available up to January 10, 2023, with collection dates spanning 2 November to 28 December 2022 (n=14), all bearing E50K (C) as defined for ECDC subgroup i (Nextstrain "2b") viruses [1]. All additionally possessed F79V + I140K (A) + G275D (C) as per viruses identified by the Canadian Sentinel Practitioner Surveillance Network (SPSN) during the 2022/23 season in British Columbia, Canada. Not displayed are ECDC clade 3C.2a1b.2a.2 viruses in other ECDC subgroups that may also bear T135K substitution.

Segment ID	Segment	Country	Collection Date (yyyy-mm-dd)	Isolate ID	Isolate Name	Originating Laboratory	Submitting Laboratory	Authors
EPI2239159	HA	United States	2022-Aug-03	EPI_ISL_16045848	A/Michigan/UOM10046751120/2022	University of Michigan Clinical Microbiology Laboratory	University of Michigan	
EPI2239175	HA	United States	2022-Aug-08	EPI_ISL_16045883	A/Michigan/UOM10046833868/2022	University of Michigan Clinical Microbiology Laboratory	University of Michigan	
EPI2248969	HA	United States	2022-Oct-08	EPI_ISL_16199248	A/Washington/56/2022	Washington State Public Health Laboratory	Centers for Disease Control and Prevention	
EPI2279434	HA	United States	2022-Oct-18	EPI_ISL_16385140	A/Washington/61/2022	Washington State Public Health Laboratory	Centers for Disease Control and Prevention	
EPI2243287	HA	Canada	2022-Oct-27	EPI_ISL_16100899	A/Saskatchewan/SKFLU279237/2022	Roy Romanow Provincial Laboratory	Row Romanow Provincial Laboratory	McDonald,Ryan; MacKenzie,Keith; Faires,Meredith; Loos,Kara; Kary,Stefani; Klassen,Laura; Lang,Amanda; Minion,Jessica; Roy Romanow Provincial Laboratory,Molecular Diagnostics
EPI2243343	HA	Canada	2022-Nov-06	EPI_ISL_16101519	A/Saskatchewan/SKFLU244207/2022	Roy Romanow Provincial Laboratory	Row Romanow Provincial Laboratory	
EPI2244274	HA	Canada	2022-Nov-23	EPI_ISL_16118279	A/Saskatchewan/SKFLU273032/2022	Roy Romanow Provincial Laboratory	Row Romanow Provincial Laboratory	
EPI2253120	HA	United States	2022-Nov-14	EPI_ISL_16201522	A/Idaho/49/2022	State of Idaho Bureau of Laboratories	Centers for Disease Control and Prevention	
EPI2279450	HA	United States	2022-Nov-15	EPI_ISL_16385142	A/Wyoming/19/2022	Wyoming Public Health Laboratory	Centers for Disease Control and Prevention	
EPI2279713	HA	United States	2022-Nov-19	EPI_ISL_16385243	A/Washington/67/2022	Washington State Public Health Laboratory	Centers for Disease Control and Prevention	
EPI2278446	HA	United States	2022-Nov-28	EPI_ISL_16384377	A/Nebraska/34/2022	Nebraska Public Health Lab	Centers for Disease Control and Prevention	
EPI2283460	HA	Denmark	2022-Nov-29	EPI_ISL_16444867	A/Denmark/3198/2022	Statens Serum Institut	Statens Serum Institute	Trebbien, Ramona; Bolt Botnen, Amanda; Ciucani, Marta
EPI2283266	HA	Denmark	2022-Nov-29	EPI_ISL_16444472	A/Denmark/3189/2022	Statens Serum Institut	Statens Serum Institute	
EPI2283838	HA	United States	2022-Dec-02	EPI_ISL_16447137	A/Wyoming/24/2022	Wyoming Public Health Laboratory	Centers for Disease Control and Prevention	

References, Supplementary Material

1. European Centre for Disease Prevention and Control (ECDC). Influenza virus characterization, summary Europe, November 2022. Stockholm: ECDC. [Accessed: 16 January 2023]. Available from: https://www.ecdc.europa.eu/en/publications-data/influenza-virus-characterization-summary-europe-november-2022

2. Nextstrain. Real-time tracking of influenza A/H3N2 evolution. [Accessed 30 January 2023]. Available from: https://nextstrain.org/flu/seasonal/h3n2/ha/2y

3. Koel BF, Burke DF, Bestebroer TM, van der Vliet S, Zondag GC, Vervaet G, et al. Substitutions near the receptor binding site determine major antigenic change during influenza virus evolution. Science. 2013;342(6161):976-9. https://doi.org/10.1126/science.1244730 PMID: 24264991

4. Shu Y, McCauley J. GISAID: Global initiative on sharing all influenza data - from vision to reality. Euro Surveill. 2017;22(13):30494. https://doi.org/10.2807/1560-7917.ES.2017.22.13.30494 PMID: 28382917

5. Skowronski DM, Leir S, Sabaiduc S, et al. Influenza vaccine effectiveness by A(H3N2) phylogenetic subcluster and prior vaccination history: 2016-2017 and 2017-2018 epidemics in Canada. J Infect Dis. 2022;225(8):1387-98

6. Skowronski DM, Sabaiduc S, Leir S, Rose C, Zou M, Murti M, et al. Paradoxical clade-and age-specific vaccine effectiveness during the 2018/19 influenza A(H3N2) epidemic in Canada: potential imprint-regulated effect of vaccine (I-REV). Euro Surveill. 2019;24(46):1900585. Doi: 10.2807/1560-7917.ES.2019.24.46.1900585. PMID: 31771709

7. Bodewes R, Mutsert G, van der Klis FRM, et al. Prevalence of antibodies against seasonal influenza A and B viruses in children in Netherlands. Clinical and Vaccine Immunology 2011;18:469-76.

8. Doll MK, Pettigrew SM, Ma J, Verma A. Effects of confounding bias in coronavirus disease 2019 (COVID-19) and influenza vaccine effectiveness test-negative designs due to correlated influenza and COVID-19 vaccination behaviors. Clin Infect Dis. 2022;75(1):e564-e571. https://doi.org/10.1093/cid/ciac234.
